# The Regulatory Role of Silicon in Mitigating Plant Nutritional Stresses

**DOI:** 10.3390/plants9121779

**Published:** 2020-12-15

**Authors:** Nusrat Ali, Elise Réthoré, Jean-Claude Yvin, Seyed Abdollah Hosseini

**Affiliations:** Centre Mondial de l’Innovation Roullier, Laboratoire de Nutrition Végétale, Pôle Stress Abiotiques, 18 avenue Franklin Roosevelt, 35400 Saint-Malo, France; Nusrat.Ali@roullier.com (N.A.); Elise.Rethore@roullier.com (E.R.); JeanClaude.Yvin@roullier.com (J.-C.Y.)

**Keywords:** plant nutrition, abiotic stress tolerance, biotechnology and soil science

## Abstract

It has been long recognized that silicon (Si) plays important roles in plant productivity by improving mineral nutrition deficiencies. Despite the fact that Si is considered as ‘quasi–essential’, the positive effect of Si has mostly been described in resistance to biotic and tolerance to abiotic stresses. During the last decade, much effort has been aimed at linking the positive effects of Si under nutrient deficiency or heavy metal toxicity (HM). These studies highlight the positive effect of Si on biomass production, by maintaining photosynthetic machinery, decreasing transpiration rate and stomatal conductance, and regulating uptake and root to shoot translocation of nutrients as well as reducing oxidative stress. The mechanisms of these inputs and the processes driving the alterations in plant adaptation to nutritional stress are, however, largely unknown. In this review, we focus on the interaction of Si and macronutrient (MaN) deficiencies or micro-nutrient (MiN) deficiency, summarizing the current knowledge in numerous research fields that can improve our understanding of the mechanisms underpinning this cross-talk. To this end, we discuss the gap in Si nutrition and propose a working model to explain the responses of individual MaN or MiN disorders and their mutual responses to Si supplementation.

## 1. Introduction

Our knowledge about the importance of mineral nutrition (MN) in agriculture dates back to the 19th century following Justus von Liebig’s discovery of the role of certain elements in plant growth and development [1]. He considered Si as an essential element, along with nitrogen (N), phosphorus (P), potassium (K), calcium, sulfur (S), magnesium (Mg) and sodium. However, based on the proposed criteria by Arnon and Stout in the 20th century, Si was not deemed to be an essential element [2]. Nevertheless, hundreds of studies have pointed to the positive effects of Si on plant growth. Epstein considered Si as a ‘quasi –essential’ element for plant growth, and by 2005 Si was accepted as an essential element for higher plants [3,4]. The pioneering work by Ma et al. [5] on the discovery of Si transporters further confirmed Si as a ‘’beneficial substance’’ [6,7].

Si is one of the most important trace elements for human health [8] and it also plays a crucial role in plant health, particularly in alleviating environmental stresses. In the last few years, research has waved to put efforts to understand the effect of Si on mitigation of abiotic and biotic stresses [9,10,11], with drought and salinity as the two most frequent stress triggers. The role of Si has also been identified as an inducer of plant defense and as an inhibitor of insect resistance to insecticide and thus a good candidate for pest management in the field [12]. Among abiotic stresses, a conclusive role of Si in alleviation of individual MN deficiencies has also been demonstrated [13,14,15]. Three domains are usually put forward to discover how Si nutrition can improve plant growth: (1) the role of Si in mitigating abiotic stresses like drought and salinity stress; (2) the role of Si in alleviating biotic stress; (3) the role of Si under MN deficiency or HM toxicity. This review will focus on the third mechanism mainly MN deficiency, whereby Si nutrition improves plant growth under different individual nutrient deficiencies. To avoid overlaps with some excellent recent reviews (for review see [16]) who discussed the controversies in Si nutrition, we do not focus on these aspects of Si nutrition. Instead, we focus on plant nutrient deficiency and toxicity and its link to Si nutrition, an emerging field that is currently undergoing rapid growth.

**Figure 1:**
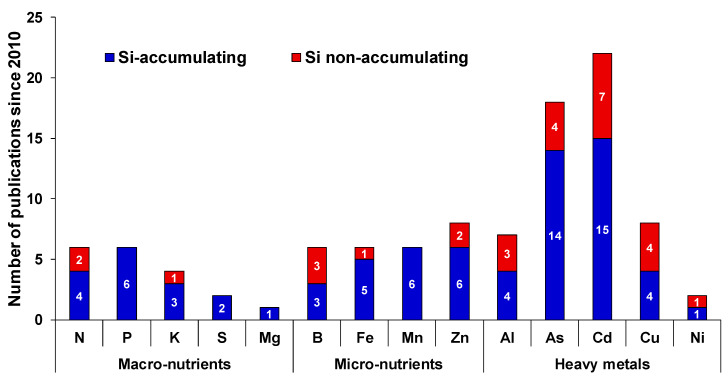
Number of major published articles indicating only the cross-talk between Si and nutritional stress since 2010. For each element, the number inside each bar indicates the number of published work in Si-accumulating (blue bar) or Si-non-accumulating species (red bar).

In this review, we aim to discuss in detail the essential nature of Si under different MaN and MiN stresses. As already known, Si impacts uptake, translocation and availability of several mineral nutrients in plants. However, the effects are not common and depends on crop species, plant capacity to accumulate Si and other interfering environmental factors. Although there is a clear discrepancy between the Si-accumulating and Si-non-accumulating plant species, it is still difficult to define the integrated role of Si as majority of studies have been reported in Si-accumulating plants (Figure 1).

**Figure 2:**
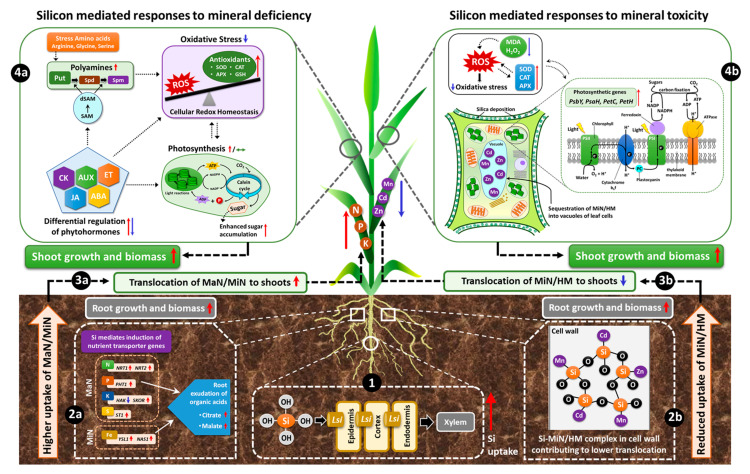
Schematic model displaying Si-mediated shared responses to nutritional stresses. The initial response to Si under nutritional stress is the uptake of supplied Si, mediated by induction of respective *Lsi* transporters in the roots (**1**). Consequently, under nutrient deficiency, Si induces the genes encoding the uptake and translocation of MaN/MiN in roots and facilitates increased uptake of the corresponding nutrient. For elements like P or Fe, root exudation of organic acids can lead to enhanced uptake of these nutrients (**2a**). Si-mediated higher uptake of nutrients further improves growth, biomass and related physiological parameters in the roots and results in increased translocation of MaN/MiN to the shoots (**3a**). In shoots, Si interacts with phytohormones, amino acids, metabolites and differentially regulates their synthesis both at the transcriptional and metabolic levels and thereby lowers oxidative stress by further modulating the antioxidant enzymes (**4a**). The simultaneous regulation of phytohormones/metabolites maintains or even induces higher photosynthetic efficiency resulting in enhanced sugar accumulation and eventual augmentation of plant growth and development and related physiological parameters (**4a**). On the other hand, for mineral/metal toxicity, Si employs a protective role by forming a complex with MiN/HM in the root cell wall (**2b**) and therefore contributes to reduced uptake and translocation of MiN/HM to the shoots (**3b**). In shoots, Si accumulation further mitigates metal toxicity by sequestration of MiN/HM into the vacuoles of leaf cells and simultaneously Si accumulation in the leaves improves photosynthetic efficiency by inducing the expression of photosynthesis-related genes (**4b**). In addition, Si reduces lipid peroxidation by lowering MDA and H_2_O_2_ levels and further increases the activity of antioxidant enzymes, which restores the redox balance, thus decreasing oxidative stress and consequently promoting plant growth and biomass (**4b**). Overall, Si has the potential of enabling the plant to react adaptively against nutritional stress responses and promotes tolerance. (Red arrows = increase/upregulation, blue arrows = decrease/down-regulation, dashed black arrows = hypothesised/proposed regulation, green double-headed arrow = maintained/preserved).

Therefore, we considered the recent efforts linking Si to MaN/MiN irrespective of plant capacity to uptake Si and have provided a working model by which different nutrients may share benefits from Si supplementation (Figure 2).

Under nutritional deficiency, Si increases the expression of MaN/MiN transporters in the root and favors the translocation of nutrients to the shoot, where it further modulates metabolic pathways and improves photosynthetic efficiency. However, under MiN or HM toxicity, Si is usually complexed with MiN/HM in the root cell wall and thereby reduces root to shoot translocation of toxic elements, while in shoots Si mitigates toxicity by sequestering MiN/HM into the leaf vacuoles and simultaneously decreases oxidative stress by maintaining redox balance.

Additionally, we also discuss the gap linking Si and mineral deficiencies/toxicities and focus on potential approaches to decipher the largely unknown role of Si. Together, these information can enhance our understanding of the interactions between Si and MN, and how these processes can be oriented in the future studies in Si field.

## 2. Si and Soil

### Si in Soil and Its Availability for Plants

Si is the second most abundant element on the earth crust but its bioavailability in soils depends on its biogeochemical cycling, its mineral form and its solubility [17]. Moreover, in the field, the pool of available Si tends to decrease, since the Si accumulated in crops are usually exported and not recycled in soil [16], which raises the question of Si fertilization. Si is present in the soil in various forms of which most types are only partially soluble. Monosilicic acid (Si(OH)_4_) is the main water-soluble form of Si [18], although it can be converted to H_3_SiO_4_^−^ at pH above 9 and to H_2_SiO_4_^2−^ above pH 11 [16]. Thus, available Si in soils includes monosilicic acid in soil solution (0.1 to 0.6 mM) and parts of silicate components that can be easily converted into monosilicic acids such as polymerized silicic acid, exchangeable silicates and part of colloidal silicates. Si is usually less present in the soil solution at high pH (8–9) because of a high adsorption of H_3_SiO_4_^−^ to soil colloids through interactions with iron and aluminium oxides [18]. However, the effect of pH on Si availability is more complex and is highly dependent on other parameters, such as soil texture, temperature, organic matter and accompanying ions [17]. Even in a condition where Si is abundant and available to plants, their capacity to accumulate silicon greatly varies from one species to another, and even from one genotype to another, within the same species [14,19,20]. The highest Si-accumulator plants are present among the Equisetales, Cyperales and Poales families, and they can accumulate Si up to 10% of shoot dry weight, such as in rice plants [21]. Some other families, such as Cucurbitales, Urticales and Commelinaceae moderately accumulate Si (2–4%) [22], while other plants do not uptake Si or exclude it [23]. These differences in plant silicon content are directly linked to the capacity of the plants to efficiently uptake Si from the soil, which is related to the expression of Si transporters in the roots [24]. Si transporters have been firstly identified in rice plants: *Lsi1* as the primary influx transporter between soil solution and root symplast and *Lsi2* as the main efflux transporter between root symplast and root apoplast [19,21]. The transporter *Lsi6* was later discovered as the transporter responsible for xylem unloading [25]. These transporters were also found in other crops such as barley and corn [25,26] and it was shown that *Lsi1* and *Lsi2* were localized to distinct cells in these plants, contrary to rice roots, in which there are present in the same cell but to opposite sides of the cell [7]. In rice, the more efficient translocation of Si allows the accumulation of 90% of the Si in the shoot [24]. It’s worthy to note that the aquaporins are also involved in the uptake and translocation of Si in few crop plants. For example, nodulin-26-like intrinsic proteins (*GmNIP2-1* and *GmNIP2-2*) have been identified as Si influx transporters in soybean compared to Si-accumulating rice plants [27]. Si is absorbed by the roots as monosilicic acid and is then precipitated inside the plant as amorphous silica inside the epidermis cell wall or in specific cells (opal and phytolith) [28]. This process is particularly helpful to protect the plant against a multitude of stresses, such as mineral deficiency or toxicity, which we will further detail in the next sections.

## 3. Si and Macro-Nutrient Deficiency

### 3.1. Si and Nitrogen Deficiency

MaNs play a crucial role in plant growth and development and irregularities in their availability can negatively impact plant biology and yield. Nitrogen (N) is one of the main elements required in large amount by plants which is an integral constituent of proteins, nucleic acids, chlorophyll, co-enzymes, phytohormones and secondary metabolites. Therefore, lack of N in soil or a decline in root uptake capacity will negatively affect plant productivity [1]. One of the areas of interest in Si research is its role in alleviation of MaN deficiencies [15,29,30]. In the last few years, research has focused on the cross-talk between Si and N nutrition. The first work considering Si and N interactions was reported in hydroponically grown rice as Si-accumulator plant. These authors showed that Si induced the expression of the genes involved in N uptake/translocation under low N supply, but that of *OsAMT1;1* and *OsGS1;1* were suppressed under high N input [31]. These authors also showed an increase in the expression of *OsNRT1:1*, *OsGS2*, *OsFd-GOGAT*, *OsNADH-GOGAT2* and *OsGDH2* under low N input. They concluded an antagonistic interaction between N and Si in rice where Si differentially changes the uptake and assimilation of N in rice plants. Another effort showing cross-talk between Si and N was on hydroponically grown rapeseed plants, which are classified as non-Si accumulators [32]. It has a high capacity to take up nitrate from soil but displays low nitrogen use efficiency (NUE) due to the lack in N remobilization to leaf [33]. Haddad et al., [32] showed that one-week pre-treatment with Si increased plant biomass, chlorophyll level and the expression level of the high-affinity nitrate transporter *BnaNRT2.1*. These authors also showed a delay in leaf senescence in N-deprived plant treated with Si, as confirmed by lower induction of senescence marker genes particularly when N was resupplied. Extending their research to the field, the same group obtained different results where the positive effect of Si on rapeseed yield and N uptake was observed but only at higher doses of N (160 kg/ha N). Comparing the above studies clearly indicates that Si modulates the two types of nitrate transporters involved in root nitrate uptake in which the low-affinity transporter *NRT1:1* was modulated in Si-accumulator rice plants and the high-affinity *NRT2.1* was modulated in non Si-accumulator rapeseed plants. Nevertheless, the reports that Si change the expression level of the N transporters is based solely on gene expression analyses and the level of N in different tissues; hence, they do not provide a clear evidence of a direct involvement of Si in this process. In the context of nitrate transporters, the role of Si in radial transport of nitrate across the root and its loading to xylem stream and later within the shoot is still obscure. Furthermore, whether and to what extent Si is involved in the uptake and transport of ammonium and inorganic N uptake like urea within plants lack evidence.

We believe that reported works on the cross-talk between Si and N could be positive evidence, but needs to be experimentally investigated in detail by taking into account further approaches such as ^15^N uptake labelling experiment or using transgenic lines and mutants to concretely demonstrate the role of Si in the uptake/translocation of N within the crop plants. Moreover, in both rapeseed trials, the authors applied the same source of N (NH_4_NO_3_) and Si (Si(OH)_4_), but, the contrasting results on the role of Si either under low or high N inputs rise the question if plants benefit from Si supplementation into the solutions or directly into the soil as fertilizer. Therefore, the response of Si under low or high N input could be due the fact that Si function can be different from experimental solution to the field condition mainly because Si chemistry in the soil is very complex [16].

Moreover, interactions of Si with other elements like carbon (C) should be further evaluated. Indeed, it was shown in *Holcus lanatus* that Si/C ratio responded to N and P status, and that the decrease in this ratio under nutrient limitation could be linked to the lower energy consumption required for Si uptake compared to C uptake [34]. Further research on the cross-talk between Si and N has also compared the sole or combined effect of Si and salicylic acid under N deficiency in rice. It was shown that N-deprived rice plants only benefit from Si when Si was solely sprayed to the leaves in which the carbon status eventually increased the lignin synthesis, which overall enhanced the yield [35]. This study lacks microscopic data and did not represent lignification at casparian strip level and concluded based on the lignin content in the tissue; however, it opens a new way to study in detail the role of Si in nutrient stresses induced suberization in casparian strips—cell wall [36]. In the roots of both rice and maize as Si-accumulator plants, Si promoted casparian strips formation [37]. Further study using transcriptomic approaches also revealed the involvement of several candidate genes in the Si-induced formation of casparian strips such as ATP binding cassette transporter, class III peroxidases, and ligases [38]. In this context, the discovery of the mutants displaying discontinuous or defection in formation of casparian strips [39] can provide an excellent opportunity to clarify the obscure function of Si in lignification or suberization of casparian strip under macronutrient deficiencies.

### 3.2. Si and Potassium Deficiency

Among essential macronutrients, potassium (K) is considered as an indispensable mineral nutrient for crop plants. It plays an essential function in central metabolic processes like photosynthesis, protein biosynthesis, osmoregulation, turgor driven movements, and maintenance of the plasma membrane potential [40]. Evidence for cross-talk between Si and K deficiency was first provided in hydroponically grown soybean by a Chinese group [15]. The authors showed that Si ameliorated oxidative stress by modulating antioxidant enzymes, had positive effect on root and shoot biomass and increased the shoot K. Looking attentively to their results, root parameters were positively improved by the application of sodium silicate (Na_2_SiO_3_) under the lack of K. However, the root responses of K-deprived plants treated with sole NaCl was similar to that of control plants, highlighting a point of caution that must be considered for selecting the source of applied Si, even if the equilibrium of Na was anticipated in the experiment. This is crucial as Na can be replaced in varying degree by K [1] and K substitution by Na has been already reported in plants like Eucalyptus seedlings [41] and wheat [42]. In another study in hydroponically grown sorghum, it was shown that Si changed the expression level of SKOR channel mediating K secretion from root cortex cells into the xylem while down-regulating the *HAK5* and *AKT1* transporters to preserve higher K in the xylem, modulating aquaporin activities and leading to enhanced root hydraulic conductance under K deficiency [43]. The authors speculated that Si plays a role in plant signaling regulation under K-deficient stress which may lead to enhanced K concentration in the xylem. Changes in the expression pattern of *SKOR* channel can be a very interesting body of Si nutrition. The regulation of xylem K plays a crucial role in the regulation of xylem hydraulic conductance and water relation in plants which might eventually lower the risk under water-deficient condition [44]. The increase in xylem K and its link to higher xylem hydraulic conductance was shown in *Laurus nobilis* [45].

It is noteworthy that K is involved in long-distance transport system in which it is required for sucrose loading into phloem [1,46]. A recent analysis, showed that K is in fact circulating in the phloem (between sieve cells and companion cells) and serves as a decentralized energy storage that can be used to overcome local energy limitations in the phloem. Phosphorylation of the expressed phloem-specific *AKT2* protein “fuels” this potassium battery, which then efficiently assists the plasma membrane H+-ATPase in energizing the transmembrane phloem sucrose (re)loading process [47]. Lack of this critical reloading of sucrose would impair the long-distance transport within plants and thus reduce plant productivity. Hence, deciphering the regulatory role of Si in modulating the *AKT2* channel in relation to the vascular circulation of sucrose would be an interesting subject in future research on cross-talk between K and Si.

In another work in sorghum, it was also shown that Si mitigated K deficiency symptoms by reducing the level of the polyamine putrescine, thus alleviating oxidative stress [48]. Recently, our group investigated the role of Si in barley grown under concomitant osmotic stress and K deficiency, using metabolite and hormonal profiling [49]. We showed that the higher induction of Si transporters and consequent increase in leaf Si was associated with sugar loading to the root and increased root biomass. We also reported that Si application affects the expression of the genes involved in abscisic acid (ABA) and cytokinin homeostasis, subsequently delaying leaf senescence. K deficiency signaling pathway is associated with different components like ROS-ethylene interaction, Ca^2+^ signaling pathway, auxin and jasmonic acid synthesis [50]. In the meantime, K is also involved in the activation of large number of enzymes associated with different metabolic pathways. Therefore, we believe that, while designing the future omics experiments, the RNAseq approach specifically needs to be taken into account. This can open the way to deeply decipher the gene networks and their relevant metabolites pathways influenced by Si supplementation, in this case under K deficiency, and thus will aid in an accurate interpretation of the results. Moreover, rubidium uptake experiment can properly verify if Si is directly involved in root K uptake and its transport to aerial part of plants or it is only the result of the induced stress.

### 3.3. Si and Phosphorus Deficiency

P is an essential macronutrient for plant growth and development. It is responsible for the strongly acidic nature of nucleic acids, and thus for the high cation concentrations in DNA and RNA and represent the metabolic energy of cells [1]. One of the areas of Si research is its interaction with P. Kostic et al. [51] demonstrated that Si increased the uptake of P in wheat grown in acidic soil associated with the up-regulation of the expression of P transporters *TaPHT1.1* and *TaPHT1.2* and increased root exudation of organic acids citrate and malate, linked to higher uptake of P. Conversely, Hu et al. [52] showed that higher accumulation of Si in rice shoots was associated with decreased P uptake via reduced expression of the root P transporter gene *OsPT6*. To our best knowledge, this research is the only attempt to consider mutant of Si transporter *OsLsi1* which yielded more reliable observation on the P uptake induced by Si application. The differences observed in the regulation of P transporters between rice and wheat crops could be attributed to the nature of the two plants to utilize Si as well as the difference in the source and doses of P. Notably, the pH of the culture/soil in this case growing wheat under acidic condition might displays additional nutritional imbalances which can eventually influence on the regulation of P transporters. We strongly believe that the mechanism underlying the regulatory role of Si on the expression of P transporters remains to be further investigated in the future. In another work, Chaiwong et al. [53] showed an increased in root biomass in two varieties of hydroponically grown rice plants in response to Si nutrition alone or in combination with iron (Fe) or P. The authors showed that Si positively increased the root biomass (39%) under concomitant P and Fe deficiencies, which were also genotype dependent responses. In their study, Si did not change the expression of P transporters *OsPHO1;2*, which is involved in translocation of P from root to shoot and the sole Si deficiency and its combination with Fe deficiency did not improve translocation of P to the shoots as supported by ^33^Pi labelling experiment.

### 3.4. Si and Magnesium or Sulfur Deficiency

Cross-talk between Si and elements like S and Mg has not been studied extensively, and recently our group was the first to assess this interaction [29,54]. We have shown that, in barley plants subjected to osmotic stress and S deficiency simultaneously, Si modulated the genes involved in S and ABA homeostasis. Even though another stress trigger (osmotic stress) combined with S deficiency, nevertheless, this pioneering work could open the new windows to study deeply the cross talk between Si and S. Therefore, in our recent work, we attempted to decipher the role of Si application solely on the metabolism of Si-accumulator rice plants under S deficiency [55]. The study demonstrated a distinct source to sink metabolic regulation coupled with changes in the expression level of S and Si transporters leading to a consequent higher accumulation of Si and lower accumulation of stress phytohormones in the shoots, therefore maintaining plant growth and development under S deprivation [49]. Similarly, linking Si to Mg deficiency, we showed that Si influences the contents of organic acids, amino acids (GABA, Gly and Ser) and modulates cytokinin, jasmonic acid (JA) and its derivative JA-Ile, to cope with the lack of Mg. In this regard, under both S and Mg deficiency, shoots showed enhanced sugar and polyamine accumulation under Si supply. Indeed, dealing with Si nutrition and its role under abiotic stresses during last few years, we found soluble sugar and polyamine augmentation most probably as a mutual response [29,54]. Cross-talk between Si and these two nutrients (Mg and S) also was shown in two forage plants where Si increased their biomass only under Mg deficiency by increasing the uptake of Mg [56]. In this context, additional experimental evidence is needed to draw a concrete conclusion. Si also appears to differentially affect different metabolic and hormonal pathways. Future works need to be focused on this aspect of Si at both biochemical and molecular levels, considering the role of Si in different type of species like Si-accumulator, intermediate and non-accumulator. This could further support insights into the underlying mechanisms by which Si promotes recovery from MaN stresses in crop plants.

## 4. Si and Micro-Nutrient Deficiency or Heavy Metal Toxicity

Numerous studies have recently been undertaken with the aim to unravel the link between Si and MiN. Apart from Fe, which is studied as a deficient MiN, the remaining work highlighting the interaction between Si and other MiNs is mainly considering them as potentially toxic elements.

Pavlovic et al. [57,58] provided the first evidence that Si alleviates Fe deficiency in cucumber by mobilization of Fe to younger leaves and root secretion of citrate and malate. Si also up-regulated the expression of Fe-related genes (*NAS1* and *YSL1*) which subsequently increased the nicotianamine levels and facilitated phloem loading–unloading of Fe. More recently, an analysis of cucumber and barley showed that Si mitigated Fe deficiency by increasing Fe content in the young leaf and redistribution of certain metals like Cu and Zn [59,60]. Noteworthy is that Si-ameliorated Fe deficiency was found to be plant-specific and pH-dependent [59,61]. However, the above-mentioned works were all performed in hydroponic conditions, but in soil the scenario could vary depending on several interactive factors. Beside soil pH, the solubility of primary and secondary minerals presents in the soil, stage of soil development and the content of weatherable silicate are the most important factors controlling Si concentration in the soil solution. In this context, silicate can be adsorbed on soil colloid surfaces by Fe and Al hydrous oxides. Due to large surface area, aluminum oxides are generally more effective than Fe [62]. Therefore, considering the extra effect of pH in Si availability in the soil, it is still unclear how effective would be Si fertilization is soil that lacks Fe [63].

During the last decade, researchers have assessed the effects of Si under excess boron (B), mainly in soil-grown cereals [64,65,66,67]. A recent study showed that Si application alleviated the harmful effects of B deficiency and toxicity in cotton by different modes of Si application [68]. The authors showed that foliar Si application was effective under B deficiency, by improving the photosynthesis-related parameters, while root application of Si restricted B transport into shoots under B toxicity. Other studies also focused on different mechanisms by which Si alleviates B toxicity, such as by reducing oxidative stress and by modulating activities of antioxidant enzymes, and by reduction in leaf B accumulation [64,69]. 

The majority of work on Si and zinc (Zn) toxicity has used hydroponically grown rice. Results showed a reduction in both root and shoot Zn content upon Si supply and changes in photosynthesis-related genes under Zn toxicity [70,71,72]. Anwaar et al. [73] similarly showed that Si mitigated Zn toxicity in cotton by reducing Zn level in both roots and shoots and reduced oxidative stress through effects on antioxidant enzymes. Despite higher Zn accumulation in maize shoots, Si reduced metal toxicity by altering the organelle structure and Si deposition in the endodermis and pericycle of roots [74]. In another study in wheat, it was shown that the root application of Si was much more effective in alleviating Zn toxicity compared to foliar application. This was due to an increase in soil pH by Si, which decreased the root accumulation of Zn and its translocation to shoot [75].

Manganese (Mn) is an essential element for the proper function of photosynthetic activity and some other enzyme activities [76]. Mn deficiency was recently shown to be alleviated by Si application in sorghum. The application of sodium and potassium silicate either to leaves or roots increased dry matter production, photosynthetic activity, manganese use efficiency and superoxide dismutase activity, although to a higher extent with root application [77]. Interestingly, the accumulation of Mn was not improved by Si application, which is in agreement with a previous work performed in cucumber [78]. This would mean that, under Mn deficiency, Si would have a rather indirect role by improving antioxidant activities, rather than directly improving Mn uptake or remobilization.

The protective role of Si against Mn toxicity has also been considered. In cucumber and cowpea, Si mitigates Mn toxicity by partitioning Mn mostly in the cell wall rather than the apoplast, so reducing Mn availability [79,80]. There are also genotypic crop-dependent differences in response to Mn toxicity upon Si supply. For example, in rice, Si reduces Mn toxicity in a sensitive genotype due to reduced translocation of Mn to the shoot, while in maize, a C4 crop, Si alleviates Mn toxicity by increasing the thickness of the epidermis, which has been considered previously as a strategy to cope with Mn stress [81,82] The lower translocation of Mn to the shoot by Si has also been shown in rice by short-term labelling experiments with ^54^Mn; in the *lsi1* mutant, the Mn–Si complex in root cells contributes to lower translocation of Mn. Reduced expression of the *OsNramp5* Mn transporter under long term Si supply reduces root to shoot translocation of Mn. Work on cucumber indicates the regulatory role of Si in reducing oxidative stress under Mn toxicity by increasing activity of antioxidant enzymes [83,84] and reducing both Mn and H_2_O_2_ levels in the apoplast.

In regard to cadmium (Cd) toxicity, rice was one of the most frequently reported crops to benefit from Si supplementation. Lower accumulation of Cd in roots and shoots and higher photosynthetic efficiency following application of Si were highlighted in rice plants exposed to Cd toxicity [85,86]. Nwugo et al. [87] identified changes in the proteome of rice following Si treatment with Cd, associated with photosynthesis, redox homeostasis, protein synthesis, chaperone activity and pathogen responses. Recently, by applying X-ray photoelectron spectroscopy in suspension cells, the co-deposition of Si-Cd in the cell wall was found, and suggested to be a reason for lower Cd uptake in rice [86]. In maize, deposition of Si in the endodermis and pericycle of root and subsequent changes in cell extensibility (Casparian bands and suberin lamellae) played major role in decreasing Cd toxicity. Si-mediated reduction in oxidative stress under Cd stress through effects on antioxidant enzymes has been reported in several other studies [88,89].

As for Zn and Cd, rice has been the predominant species for studying the link between Si and arsenic (As). Similarly, Si was reported to reduce the uptake and translocation of As to the shoot. Using the *lsi1* mutant, Sanglard et al. [90] showed that Si ameliorated the effects of As toxicity in rice in a time and genotype-dependent manner, by rescuing damage to the photosynthetic machinery. Guo et al. [91] showed that, in rice, Si and As use the same transport pathway (*Lsi1*), and Si reduced As uptake. It has also been reported that Si-mediated reduction of As level is associated with a higher uptake of methylated As in root, mediated by *Lsi1* [91]. At the same time, a study in tomato showed that Si has no effect on As concentration in roots and shoots, further suggesting that the effects are cultivar-dependent, comprising As-excluder or -accumulator genotypes [92]. The correlation between As accumulation and radial oxygen loss (ROL) has been also shown in rice plants in which plants with high ROL accumulated lower As [93]. Wu et al. [93] showed that Si supply decreased the inorganic As in shoots of both low and high ROL, but this was more pronounced in higher ROL genotypes, indicating the possible impact of Si in reducing the toxicity effect of As in cultivated rice in As contaminated soil.

The body of scientific evidence linking Si with MiN or HM stresses is relatively large compared to MaN (Figure 1 and Figure 2). Our analysis of links between Si and MiN/HM show that, in general, Si supplementation is predominantly beneficial for any given MiN/HM stress. Here, we noticed two general features of Si in relation to MiN/HM stress: the first is its capacity to reduce oxidative stress imposed by MiN/HM in different species, and the second is co-deposition of Si with these elements in the cell wall to reduce intracellular availability. However, we believe for both features, there is lack of scientific evidence, and transgenic approaches need to be considered to decipher precisely and in detail the mechanism(s) by which Si mitigates MiN/HM stress responses. Another area of focus could be to study the degree to which Si contributes to alleviate MiN deficiency/toxicity by changing the endodermal suberization. In this context, it is worthy to note that Barberon et al., [34] showed the adaptation of Arabidopsis root, which is regulated by nutrient-induced plasticity of endodermal differentiation. Their findings revealed that suberization is delayed upon germination in Fe-, Mn-, and Zn-deficient conditions and suberin lamellae deposition is highly plastic and responds to different nutritional stresses. Meanwhile, Fleck et al., [35] showed the function of Si in promoting the casparian band due to decrease in esterified phenolic compounds in both Si-accumulating and Si-excluding species. Therefore, it would be interesting to further explore this within and across species.

## 5. Remark and Prospective

**Figure 3:**
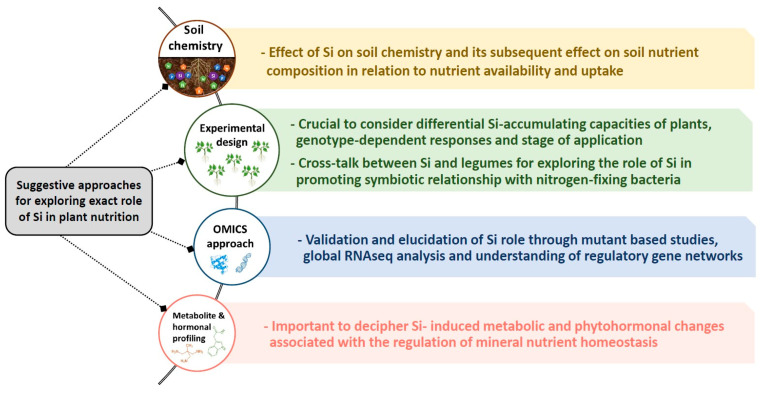
Proposed approaches for discovering the gap of knowledge in Si nutrition and its interaction with nutritional stresses.

Our review shows a clear role for Si under nutritional stress or heavy metal toxicity. In spite of numerous studies linking Si and MN, the huge gap in this field remains its exact role, which could be divided in the following (Figure 3); (a) soil: It is striking that the impact of Si on soil and its component has been scarcely investigated. This is crucial due to the importance of soil and its interaction with MN for future sustainable agriculture. There is no doubt that Si chemistry is complex due to certain soil influencing factors such as pH, texture, and organic matter [16,94], which need to be further explored experimentally. Supplying soil with Si might bring an array of changes in soil physico-chemistry and may influence soil nutrient composition. This is basically due to alkalinisation of soil pH because of the type of silicates used in different experiments and the fact that even the freely available form of Si monosilicic acid might be soluble up to a certain concentration [5]. (b) Experimental design: obviously, there is no strong consensus and unique protocol to use Si experimentally in different studies. One of the crucial points dealing with Si nutrition is classification of crop plants in regard to their ability to accumulate Si [22]. For this purpose, the different responses of Si-accumulating and non-accumulating species must be carefully considered when exposed to individual mineral stress, to build a general conclusion. Notably, as proposed by Putra et al. [95] an extensive investigation needs to be considered on the cross-talk between Si and legumes to decipher how Si promotes the symbiotic relationship with nitrogen-fixing bacteria. This body of Si is crucial in the future works as numerous studies showed the positive interactive effect of Si and plant growth promoting rhizobacteria on plant growth particularly under unfavorable environmental conditions [96]. In addition, among the same species, research on genotype-dependent response to Si nutrition need to be considered. Moreover, the optimum dose of Si as well as the stage-dependent application of Si seems an important criteria to build a precise conclusion. Although the effect of Si is more pronounced under stress conditions, a close look at the studies linking Si with MN, and Si’s role under stress-free conditions, is important. For instance, Jang et al. [97] observed a dose-dependent regulation of N and Ca^2+^ in hydroponically grown rice treated with Si, suggesting a possible role for Si even under control conditions. (c) Omic approaches: One of the key aspects of Si in various reports is its role in modulating the uptake and translocation of MN, which is difficult to explain due to the lack of understanding of a direct role for Si in plants. Although several candidate genes impacted by Si were shown, further confirmatory tests with mutants of these genes have not been reported and therefore the functionality of the genes in response to Si nutrient remains to be demonstrated. The majority of the gene expression analysis is based on up- or down-regulation in their transcript, and failed to concretely interpret if Si is directly involved in these changes or they are the consequence of the applied stresses. Therefore, this feature of Si needs also to be investigated using uptake labeling experiments, which are crucial in linking Si and its role in uptake/translocation of minerals. In the meantime, RNAseq analysis can open a new window and global understanding in the regulation of gene networks by Si supplement under mineral nutrient deficiencies considering Si-accumulating and non-accumulating species and genotype effects. (d) Metabolite and hormonal profiling; metabolites and hormones are both vital for regulation of plant nutrition homeostasis. During the last few years, our research studies provided some evidence in this regard, indicating the impact of Si nutrition under K [49], S [54,55] and Mg [29] deficiencies. Moreover, the role of Si in metabolic changes under arsenic [98] as well as the change in amino acid levels in rice has been previously reported [99]. In this context, the lack of sufficient results led to debated interpretation, and thus this cross-talk needs to be further experimentally investigated.

## Abbreviations

MN	Mineral nutrition
HM	Heavy metal
MaN	Macro-nutrient
MiN	Micro-nutrient
